# Fovea sparing internal limiting membrane peeling using multiple parafoveal curvilinear peels for myopic foveoschisis: technique and outcome

**DOI:** 10.1186/s12886-016-0356-4

**Published:** 2016-10-18

**Authors:** Haiying Jin, Qi Zhang, Peiquan Zhao

**Affiliations:** Department of Ophthalmology, Xinhua Hospital, Shanghai Jiaotong University School of Medicine, Shanghai, 200092 China

**Keywords:** Fovea, Internal limiting membrane, Myopic foveoschisis, Surgical technique, Vitrectomy, Internal limiting membrane peeling

## Abstract

**Background:**

To introduce a modified surgical technique, the “parafoveal multiple curvelinear internal limiting membrane (ILM) peeling”, to preserve epi-foveal ILM in myopic foveoschisis surgery.

**Methods:**

Consecutive patients with myopic foveoschisis were enrolled in the present prospective interventional case series. The surgeries were performed using transconjunctival 23-gauge system. The macular area was divided into quadrants. ILM was peeled off in a curvilinear manner centered around the site that was away from the central fovea in each quadrant. Shearing forces were used to control the direction to keep the peeling away from central fovea. ILM at central fovea of about 500 to 1000 μm was preserved by this technique.

**Results:**

This technique was performed in 20 eyes of 20 consecutive patients. Epi-foveal ILM was successfully preserved in all cases using the technique. Patients were followed up for more than 12 months. The mean postoperative logMAR visual acuity improved from 1.67 ± 0.65 preoperatively to 1.15 ± 0.49 (*P* = 0.015; paired *t*-test). Postoperative OCT examinations showed that full-thickness macular holes (MHs) did not developed in any case. Central fovea thickness decreased from 910 ± 261 μm preoperatively to 125 ± 85 postoperatively (*P* = 0.001; paired *t*-test).

**Conclusion:**

Fovea sparing ILM peeling using multiple parafoveal curvilinear peels prevents the development of postoperative full-thickness MHs in eyes with myopic foveoschisis.

**Electronic supplementary material:**

The online version of this article (doi:10.1186/s12886-016-0356-4) contains supplementary material, which is available to authorized users.

## Introduction

Myopic fovealschisis is a major cause of visual impairment in eyes with pathologic myopia [1–3]. As vitreous and ILM traction plays a major role in Myopic fovealschisis, vitrectomy and ILM peeling combine with gas tamponade was reported to be an effective approach in the management of the disease. Although studies have shown that ILM peeling results in long-term favorable anatomical and visual outcomes [4–6], a high risk of the postoperative development of full-thickness macular hole (MH) and retinal detachment was observed after myopic foveoschisis surgery [7–11]. The risks of MH after ILM peeling were reported to be 16.7 and 20.8 %, respectively [8, 11]. Peeling the ILM off from the fovea could induce a break of the thinned central foveal tissue and anatomical changes of the macula including dimpling or damage of the inner retina. To address this, Ho [7] and Shimada [8] proposed preserving the epi-foveal ILM during ILM peeling. This method has the advantages of achieving better anatomical and visual results over total peeling, and prevents long-term foveal retinal thinning, preventing postoperative development of full-thickness MH [7–9]. The lower rate of full-thickness MH development in myopic foveoschisis [7–10] is hypothesized to be due to the release of macular traction and reduced surgical trauma at the fovea. Nevertheless, surgical maneuver to preserve epi-foveal ILM was not a simple manipulation, especially for highly myopic eyes with long axial length. We report a modified surgical technique to preserve epi-foveal ILM, named “fovea sparing ILM peeling using multiple parafoveal curvilinear peels”.

## Methods

The present prospective study adhered to the tenets of the Declaration of Helsinki and was approved by institution review board of Xinhua hospital affiliated to medical college, Shanghai Jiaotong University. All participants provided written informed consent of possible benefits and risks. Patients undergoing myopic foveoschisis surgery by one surgeon (P.Q.Z.) from December 2012 to January 2014 were enrolled in the study. Inclusion criteria were preoperative spherical equivalent higher than-8.00 diopters (D) or axial length (AL) longer than 26 mm with myopic foveoschisis; and the recent decrease in visual function was caused by foveoschisis. Eyes with poor visual acuity due to diffuse macular chorioretinal atrophy or large Fuchs spots were excluded. Exclusion criteria also included eyes with full-thickness MH, myopic choroidal neovascularization, eyes with a history of ocular trauma and those with other retinal diseases that could affect the vision, eyes with dense opacities of the media such as corneal opacities or dense cataracts. Preoperative data collected included the age, gender, eye, best corrected visual acuity and optical coherence tomography. AL was measured by optical biometer (Ver 5.4) (Carl Zeiss Meditec AG, Jena, Germany). Follow-up includes corrected visual acuity, intraocular pressure, slitlamp examination, indirect ophthalmoscopy. OCT (RTVue-100, Optovue Inc, Fremont,CA,USA) was used to assess the preoperative and postoperative macula. Radial lines (12 lines) were used to scan the macula. Radial lines comparison report was used to identify the changes of macula before and after surgery.

## Surgical technique

**Fig. 1:**
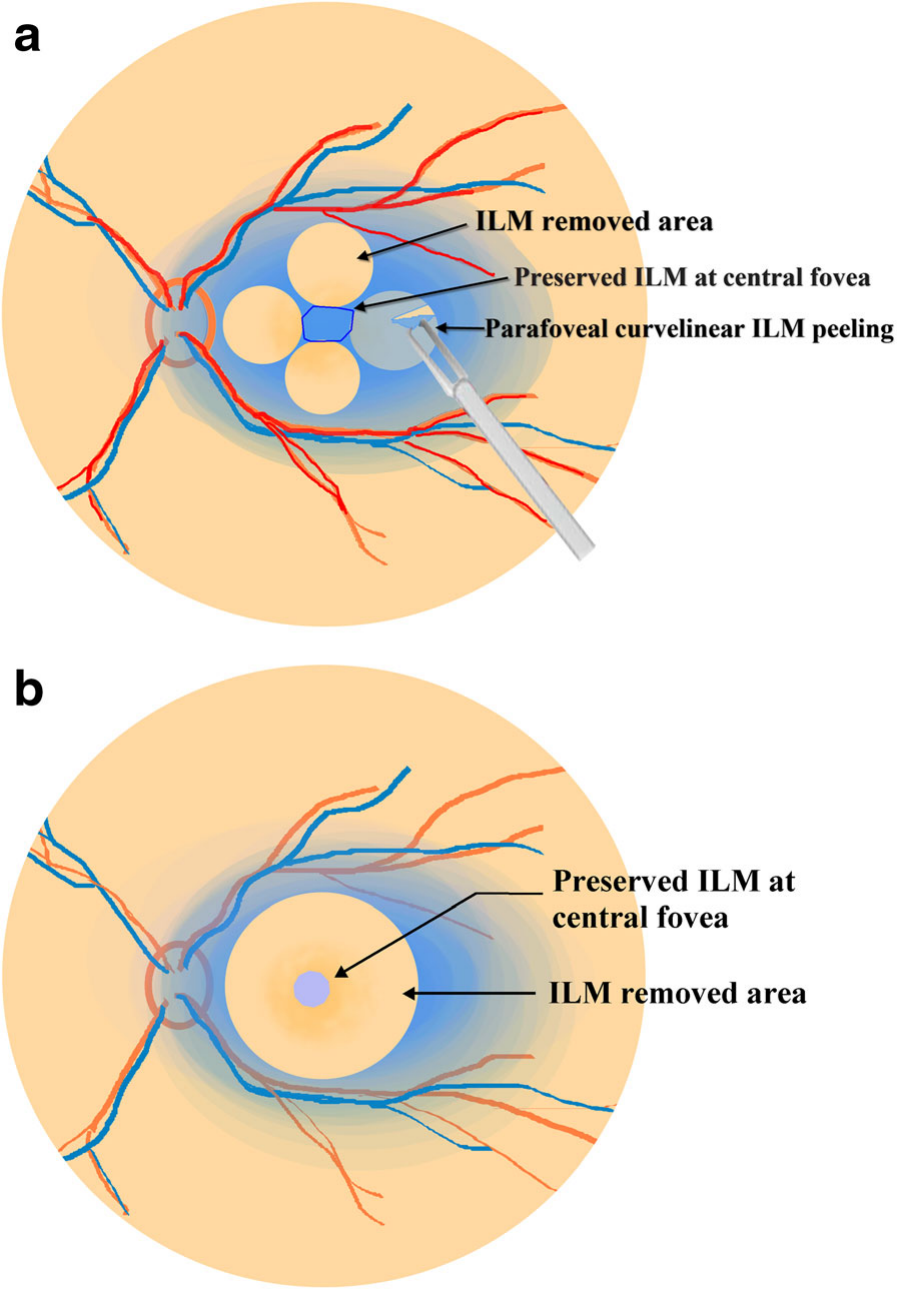
Schematic illustration demonstrating the surgical procedure of parafoveal multiple parafoveal curvelinear ILM peeling technique. **a** After the macular area was divided into quadrants, nasal, temporal, superior and inferior, ILM peeling was initiated and centered away from the central fovea in a continuous curvilinear manner in each quadrant. **b** After the parafoveal curvilinear ILM peeling, small areas of residual ILM between the four circular areas of ILM were removed. Epi-foveloar ILM of about 500 μm was preserved after multiple parafoveal curvelinear ILM peeling

**Fig. 2:**
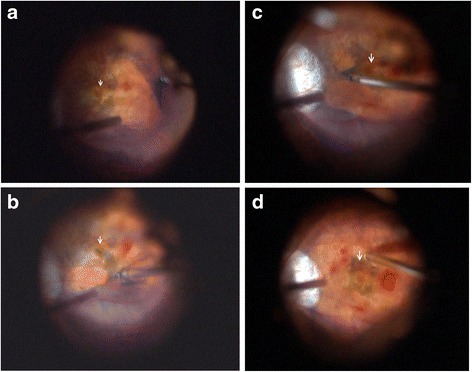
Intraoperative fundus image from surgery using the BIOM lens system (Resight 700, Zeiss, Germany). Arrows show the position of the central fovea. **a** ILM peeling started in the temporal quadrant of macula. **b**. ILM peeling in the inferior quadrant of macula. **c**. ILM peeling in the nasal quadrant of macula. **d**. Preserved ILM at the central fovea after trimming of the edge of the epi-foveal ILM using a vitreous cutter


A standard 3-port, 23-gauge transconjunctival pars plana vitrectomy was performed on all eyes under local anesthesia. After removal of core vitreous, posterior hyaloid was removed by active suction using a vitreous cutter. ILM was then stained by brilliant blue G (BBG) followed by its removal within 30 s. The macular area was divided into quadrants, nasal, temporal, superior and inferior. Initial ILM tear was performed to make a flap away from the central fovea in one quadrant using a microforceps (Grieshaber, Alcon, Fort Worth, Tex., USA). ILM was then peeled in a curvilinear manner centered around the site that was away from the central fovea in this quadrant. Shearing forces similar to that in capsulorhexis were emphasized to control the direction to keep the peeling away from central fovea. Similar maneuvers were performed to remove the parafoveal ILM in the other quadrants. After the parafoveal curvilinear ILM peeling, small areas of residual ILM between the four circular areas of ILM were removed. The edge of the residual ILM at the central fovea was trimmed by a vitreous cutter to preserve epi-foveal ILM of about 500 μm (Figs. 1 and 2). A gas tamponade (octafluoropropane) was used in all eyes. The surgical technique was named as “parafoveal multiple curvelinear ILM peeling” (demonstrated by a video in the Additional file 1). The main outcome measures were OCT findings and the best-corrected visual acuity (BCVA). All patients were followed up for more than 12 months.

Additional file 1: “Parafoveal multiple curvelinear internal limiting membrane peeling technique in myopic foveoschisis surgery”. (MP4 7616 kb)


Statistical analysis was performed using SPSS for Windows software (version 15.0, SPSS Inc.). Preoperative and postoperative central fovea thicknesses and logMAR visual acuities were compared by paired samples *t*-tests. Statistical *p* values of 0.05 or less were considered statistically significant.

## Results

**Fig. 3:**
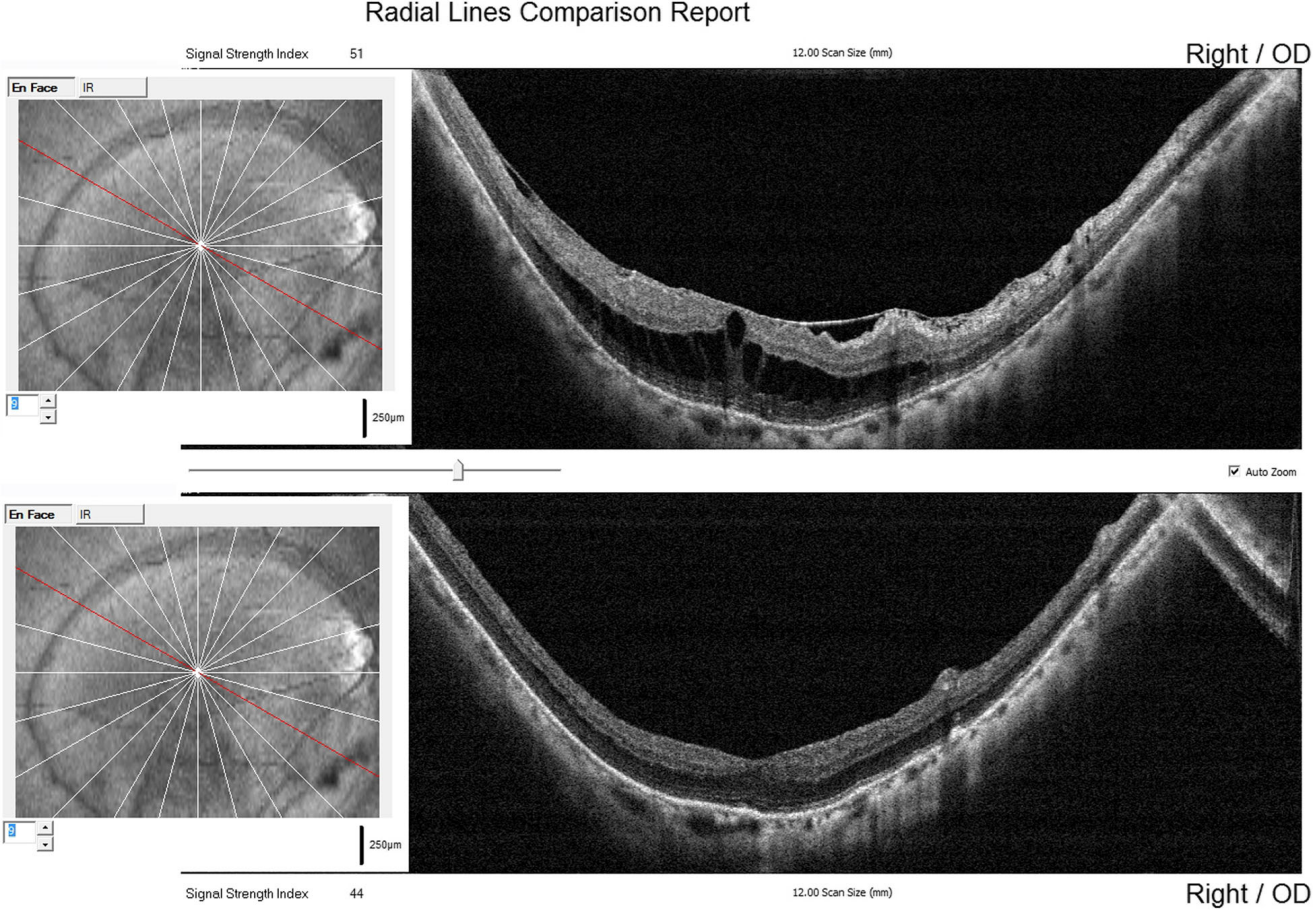
Radial lines comparison report of OCT images (12 lines) before and after surgery. Superior: preoperative image shows myopic foveoschisis; Inferior: postoperative image 12 months after fovea sparing ILM peeling. The foveal foveoschisis has been resolved with restored foveal contour. Red lines of the fundus photographs on the left indicate the same meridians of OCT scan lines before and after surgery

This technique was employed in 20 eyes of 20 consecutive patients (11 women and 6 men) with myopic foveoschisis. Mean age was 41.8 ± 7.9 years. The mean preoperative refractive error was −15.43 ± 2.98 diopters spherical equivalent. Mean axial length was 28.67 ± 2.78 mm. Average follow-up was 14.76 ± 2.35 months. Epi-foveal ILM was successfully preserved in all cases using the parafoveal multiple curvelinear ILM peeling technique. Intraoperative or postoperative vision threatening complications did not occur in the 20 cases. Mean logMAR best-corrected visual acuity improved from 1.67 ± 0.65 preoperatively to 1.15 ± 0.49 postoperatively (*P* = 0.015; paired *t*-test). At the 12-month follow-up, postoperative OCT examination showed foveoschisis were resolved in 12 eyes (60 %) and reduced in 8 eyes (40 %). Central fovea thickness decreased from 910 ± 261 μm preoperatively to 125 ± 85 μm postoperatively with restored foveal contours (*P* = 0.001; paired *t*-test) (Fig. 3). Full-thickness postoperative MHs and retinal detachment were not observed at the latest follow-up in any case.

## Discussion

ILM peeling is a widely-performed technique to manage idiopathic MH and myopic traction maculopathy. A number of studies have shown that ILM peeling results in long-term favorable anatomical and visual outcomes [4–6], However, there have been contradictory opinions published regarding total macular ILM peeling, with concerns raised regarding the anatomical changes that occur in the macula including dimpling or damage of the inner retina and a high risk of the postoperative development of full-thickness MH and retinal detachment [7–9]. The authors hypothesized that the peeling of the ILM off the fovea could induce a break in the thinned central foveal tissue. The technique of ILM peeling to preserve epi-foveal ILM was therefore advocated. Subsequent research has shown that preservation of the epi-foveal ILM has the advantages of achieving better anatomical and visual results than total peeling, and prevents long-term foveal retinal thinning, preventing postoperative development of full-thickness MH [9]. Moreover, preserving the foveal ILM was reported to prevent inner retinal damage, achieving better foveal microstructures and leading to better final visual acuity in stage 2 idiopathic MHs [10].

Two surgical techniques to preserve epi-foveal ILM was reported by different authors. One was reported by Ho and coauthors using microscissors to make multiple tangential cuts around the fovea during ILM peeling, was named the fovea non-peeling technique [7]; the other was named fovea-sparing ILM peeling described by Shimada [8]. ILM peeling was started away from the central fovea and restarted from a new site when the peeled ILM flap came close to the central fovea. Both methods performed the ILM peeling centered around the fovea. In contrast, our modified technique does not center around the fovea. Since the ultimate goal is to remove parafoveal ILM while preserving the more central foveal ILM, our methods utilized peeling that was initiated and centered away from the region of ILM we wish to remain undisturbed. We directed shearing forces to optimize peeling without lifting or removing the ILM overlying the foveal center. With the removal of the ILM in the parafoveal quadrants, the remaining epi-foveal ILM can be trimmed by a vitreous cutter. Results showed that epi-foveal ILM was successfully preserved in all cases in the present study with this technique without subsequent surgical complications. Full-thickness MH was not observed at the latest follow-up in any case. This result was identical to the previous studies performed by Ho and Shimada [7, 8]. In their studies, full-thickness MH and relevant complications were not observed in myopic foveoschisis after fovea sparing ILM peeling.

## Conclusion

The modified fovea sparing ILM peeling of using multiple parafoveal curvilinear peels was feasible and prevents the development of postoperative full-thickness MHs in eyes with myopic foveoschisis.

## Abbreviations

BBG: Brilliant blue G
ILM: Internal limiting membrane
logMAR: Logarithm of the minimum angle of resolution
MH: Macular hole
OCT: Optical coherence tomography
PPV: Pars plana vitrectomy
